# Comparison between conducted healing and the use of skin grafts for the treatment of skin wounds in rabbits

**Published:** 2008-08-15

**Authors:** Ivan Salgado Mauro, Petroianu Andy, Lelis Burgarelli Giselle, José Afonso Barbosa Alfredo, Ronaldo Alberti Luiz

**Affiliations:** * Department of Surgery from Medical School of the Federal University of Minas Gerais

**Keywords:** Wound healing, Skin graft, Skin transplant, Surgical débridement, Dressings

## Abstract

**Background:** Improvement of the healing process to provide better aesthetical and functional results continues to be a surgical challenge. This study compared the treatment of skin wounds by means of conducted healing (an original method of treatment by secondary healing) and by the use of autogenous skin grafts.

**Material and Methods:** Two skin segments, one on each side of the dorsum, were removed from 17 rabbits. The side that served as a graft donor site was left open as to undergo conducted healing (A) and was submitted only to débridement and local care with dressings. The skin removed from the side mentioned above was implanted as a graft (B) to cover the wound on the other side. Thus, each animal received the two types of treatment on its dorsum (A and B). The rabbits were divided into two groups according to the size of the wounds: Group 1 - A and B (4cm2) and Group 2 - A and B (25cm2). The healing time was 19 days for Group 1 and 35 days for Group 2. The final macro- and microscopic aspects of the healing process were analyzed comparatively among all subgroups. The presence of inflammatory cells, epidermal cysts and of giant cells was evaluated.

**Results:** No macroscopic or microscopic differences were observed while comparing the wounds that underwent conducted healing and those in which grafting was employed, although the wounds submitted to conducted healing healed more rapidly.

**Conclusion:** Conducted wound healing was effective for the treatment of skin wounds.

## Introduction

Wounds require special care for adequate healing. This may involve time and different surgical alternatives depending on the etiology and characteristics of the wound [**1**-**5**]. The longer the healing time is, the higher the risks of complications. For a wound to heal, the organism must trigger a complex process of response to trauma.

The healing phenomena, although well studied, for the most part continue to be poorly understood. There are three types of healing [**6**,**7**]:

1. The healing process *per se*, which is the ability of the organism to reconstruct itself. This process may be accelerated by débridement [**8**,**9**], cleansing of the wound and the use of sutures **10**,**11**. This process is divided into:

- Healing by first intention, which occurs when the wounds edges are approximated immediately.

- Healing by second intention, which occurs when the wound remains open for spontaneous closure. 

2. The skin grafts or flaps are used to reduce healing time, prevent infection or provide more desirable aesthetic results [**12**-**15**]. These can be divided into:

- Split-thickness skin grafts: these consist of the entire epidermis and a dermal component of variable thickness. Split-thickness skin grafts can be further categorized as thin, intermediate, or thick, based on the thickness of graft harvested.

- Full-thickness skin graft: contains epidermis, the entire thickness of the dermis, hair follicles, sebaceous and sudoriferous glands, and vessels.

3. Skin flaps consist of the entire epidermis, dermis and subcutaneous tissue and may be partially sectioned and transposed to cover a damaged area [**16**-**19**].

In our surgical experience, we observed that small wounds can heal properly with only the use of dressings and programmed débridement. The aesthetic aspect of these scars was superior when compared to the ones resulting from the use of skin grafts. This observation raised questions regarding the efficacy of wound healing without the use of skin grafts. The purpose of this work was to verify the effectiveness of the healing process of skin wounds by means of conducted healing (an original method of treatment by secondary healing) and to compare its macro and microscopically aspects to those treated with skin grafts.

## Materials and methods

This study was conducted according to the ethical principles for animal research proposed by the Ethics Committee for Animal Experimentation of The Federal University of Minas Gerais and was approved by the Ethics Committee of the Department of Surgery.

Seventeen male California rabbits aged 2.5 months with mean weights of 2088 ± 183g for group 1 and of 2567 ± 78g for group 2 were used. All animals were identified and housed in individual cages with regular food and water *ad libitum*.

The animals were operated under general anesthesia with ketamine hydrochloride injected into the gluteal region (50mg/kg). Anesthesia was complemented with lidocaine hydrochloride injected into the skin at the sites of the surgical procedure **20**.

The rabbits were divided into two groups:

- Group 1: (n = 17) a 4cm2 full-thickness skin fragment was removed from the right side of the dorsum and a second full-thickness skin segment of the same dimensions was then removed from the left side of the dorsum. The skin graft removed from the right side was placed on the wound of the left side and sutured into place. Electrocauthery was not used. Three folded gauzes soaked in liquid vaseline were used to cover the graft as a tie-over dressing and were kept in place with simple silk sutures. A similar dressing was used to cover the open wound on the right side of the dorsum. The animals of this group were followed up during 19 days, at the end of which all wounds were completely healed. Therefore, each animal had the two kinds of treatment for skin wound on its back: 

• Subgroup 1A: conducted healing.

• Subgroup 1B: grafting.

- Group 2: (n = 7) the same methods of wound treatment described for Group 1 were used to care for two 25cm2 wounds created on both sides of the rabbits dorsum . These animals were randomly selected from the rabbits from Group 1 after their wounds were completely healed (19 days). The animals from Group 2 were followed up during 35 days, at the end of which all wounds were completely healed:

• Subgroup 2A: conducted healing.

• Subgroup 2B: grafting.

The wounds treated with conducted healing were called “A” and those treated with grafts were called “B”.

The tie-over dressings were removed after three days. This method is original because of the special care of the scars. The scabs that had formed on the areas undergoing conducted healing were first removed after seven days with saline solution. This cleansing process was then repeated every three days. This kind of care improved the epithelization of the wound and prevented scar contraction. Thus, conducted healing improves wound closure and helps avoid complications such as infections or retractions.

Skin biopsies were obtained from three different areas: one from the wounds submitted to conducted healing, the other from the areas that were grafted and the third from the intact skin of the dorsum. The biopsy specimens were removed from the animals from Group1 nineteen days after their surgeries. The rabbits from Group 2 were submitted to skin biopsies 35 days after their operations. The tissue fragments were processed and stained with haematoxylin and eosin for histological analysis.

The Kruskal-Wallis test was employed to compare the measurements obtained of the tissue fragments removed from the intact epidermis, the areas that underwent conducted healing and those that were grafted observed in ten microscopic fields for each animal. The Fisher exact test was used to compare the differences among the groups regarding the presence and number of inflammatory cells, epidermal cysts and giant cells. The Wilcoxon test was used to compare the weights of the animals at the beginning and the end of the experiment. This test was also employed to compare the time necessary for the complete healing of the wounds in Group 1 and Group 2. Data was considered significant for p < 0.05.

## Results

All the animals recovered spontaneously from surgery, had uneventful follow-up and survived the experiment. The weight of the rabbits increased during the experiment period (p < 0.001 for Group 1 and p = 0.022 for Group 2) although no overweight animal was registered.

**Fig.1:**
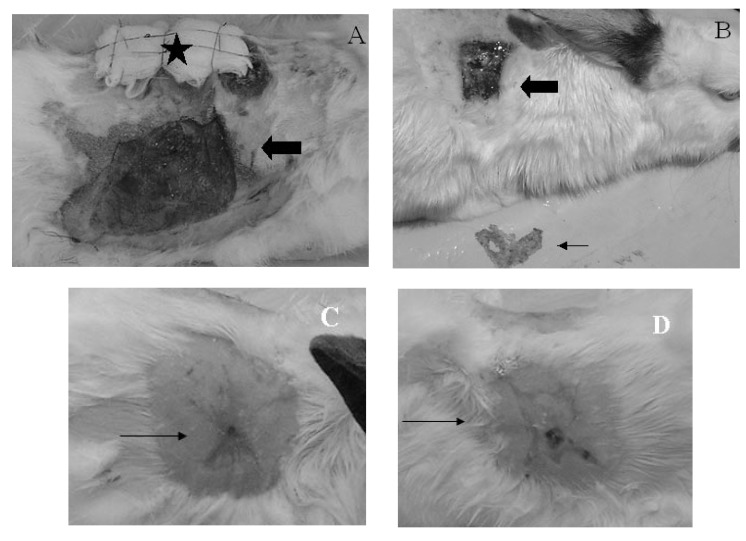
Graft and conducted healing on a rabbit of group 2. A. Conducted healing (arrow) and graft covered with a tie-over dressing (*). B. Conducted healing (large arrow) and removed scab (strait arrow) after seven days. C. Conducted healing (arrow) showing complete skin regeneration with signs of contraction and circumscriptive radial extremities (35 days). D. Skin graft (arrow) with a clear scar and open radial extremities (35 days)

The time necessary for the wounds to heal completely was defined in a pilot study – 19 days for the smaller wounds and 35 days for the larger ones. In both groups, the wounds that healed more rapidly were those submitted to conduct healing. Healing time was shorter in subgroup 1A when compared to that in subgroup 1B (p < 0.001). Subgroup 2A also tended to heal within a shorter time than subgroup 2B (p < 0.059) (**Fig. 1**).

The epidermis was moderately thickened, with well-differentiated cells, especially in the wounds treated with conducted healing. Proliferation of fibrous tissue was detected, with rare inflammatory cells. Giant cells with or without foreign bodies were detected inside the scar tissue. An inflammatory reaction was observed only in group 1. Fibrosis was also a common finding with similar distribution in all sections.

The epidermis of the skin fragments removed from the areas that underwent conducted healing and those that were grafted was thicker than that of the fragment removed from the area that was not operated upon (p<0.001 for Group 1 and p=0.03 for Group 2). The epidermal thickness did not differ among the animals that underwent surgeries.

There was a distinction regarding the presence of focal points of inflammatory cells (p=0.025) when comparing subgroups 1A and 1B (p=0.025). This was also observed in subgroups 1B and 2B (p=0.014). Epidermal cysts were more frequent in subgroup 1A (47.1%) than in subgroup 1B (11.8%) (p=0.057).

## Discussion

This work proposes a new technique for the treatment of surgical wounds – conducted healing. It involves débridement with removal of scabs at regular intervals of three days, using appropriate surgical instrumentals. Correct manipulation of the wound prevents infection, reduces healing time and changes the final aspect of the scar. The removal of scabs improved the recovery of the areas that underwent surgical procedures. New scabs that formed were successively removed until the wound was completely healed.

A variation between the initial and final weights of the rabbits was verified in both experimental groups. The similar increasing in weight of all groups shows that the animals were adequately fed. It is important to stress that the operative procedures does not seem to affect the healing process. Therefore, weight gain and adequate care of the animals must have contributed to the satisfactory healing of the wounds.

Macroscopically, the wounds submitted to conducted healing healed more rapidly than the grafted ones. Conducted healing had provided full epithelization and optimal aesthetic results. The final sizes of the grafts were smaller than their original size. Therefore, the growth of the epithelial tissue is more adequate when the wounds are left open. Epithelization of the wounds that underwent conducted healing was also more rapid than the one with graft. As was expected, the healing time of the surgical wound depends on its size, hence the wounds of group 2 took longer to heal completely than the wounds of group 1. Several studies show the importance of wound protection with the use of dressings for initial treatment [**21**-**24**,**15**]. Experiments evaluating the use of dressings for infected wounds revealed that wounds that were covered healed more rapidly than those that were left open [**25**].

**Fig.2:**
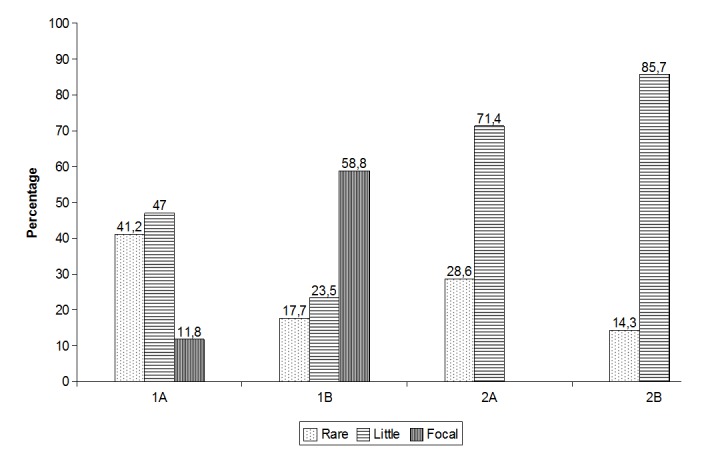
Presence of inflammatory cells - comparison among groups. Subgroup 1A – conducted healing assessment after 19 days; Subgroup 1B – skin graft assessment after 19 days; Subgroup 2A - conducted healing assessment after 35 days; Subgroup 2B - skin graft assessment after 35 days

Inflammatory cells were found only in group 1 probably because the wounds were still in an initial phase of healing. In group 2, the wounds were in a more advanced phase of healing. It should be stressed that conducted wound healing is based on skin contractility. This phenomenon is different from the contracture of abnormal scars provoked by retraction or immobility (**Fig. 2**).

This original procedure provides an option for the treatment of wounds using dressings and débridement when skin grafting is difficult to be performed. Conducted healing may eventually be adopted to avoid new wounds caused by the harvesting of skin grafts, especially in patients who are difficult to manage and require rapid operations.

## Conclusion

Conducted wound healing is effective for the treatment of skin wounds. This is an alternative for the treatment of skin wounds of different sizes and its results are as good as those obtained when grafts are used in rabbits.

## Acknowledgment

The authors gratefully thank the National Council of Science and Technology (CNPq) and the Foundation for Assistance to Research of Minas Gerais State (FAPEMIG) for the financial support.
